# A novel anti-membrane CD30 single-chain variable fragment discovered from the human phage library: A potential targeted immunotherapy

**DOI:** 10.1371/journal.pone.0284708

**Published:** 2023-04-20

**Authors:** Thanida Chanpong, Watee Seesuay, Wararat Chiangjong, Piamsiri Jiramornimit, Sarinthip Preedagasamzin, Korakot Atjanasuppat, Bunyada Jittorntrum, Somsak Prasongtanakij, Supannikar Tawinwung, Sulada Pukiat, Chonticha Saisawang, Suparerk Borwornpinyo, Khanit Sa-ngiamsuntorn, Wanpen Chaichumpa, Suradej Hongeng, Usanarat Anurathapan

**Affiliations:** 1 Graduate Program in Molecular Medicine, Faculty of Science, Mahidol University, Bangkok, Thailand; 2 Center of Research Excellence on Therapeutic Proteins and Antibody Engineering, Department of Parasitology, Faculty of Medicine Siriraj Hospital, Mahidol University, Bangkok, Thailand; 3 Department of Pediatrics, Faculty of Medicine Ramathibodi Hospital, Mahidol University, Bangkok, Thailand; 4 Office of Research, Academic Affairs and Innovation, Faculty of Medicine Ramathibodi Hospital, Mahidol University, Bangkok, Thailand; 5 Department of Pharmacology and Physiology, Faculty of Pharmaceutical Sciences, Chulalongkorn University, Bangkok, Thailand; 6 Department of Medicine, Faculty of Medicine Ramathibodi Hospital, Mahidol University, Bangkok, Thailand; 7 Molecular Medical Biosciences Cluster, Institute of Molecular Biosciences, Mahidol University, Nakhon Pathom, Thailand; 8 Department of Biotechnology, Faculty of Science, Mahidol University, Bangkok, Thailand; 9 Department of Biochemistry, Faculty of Pharmacy, Mahidol University, Bangkok, Thailand; Alfred I DuPont Hospital for Children, UNITED STATES

## Abstract

Hodgkin’s lymphoma and anaplastic large cell lymphoma, especially relapsed or refractory diseases, could recently be cured by CD30-targeted immunotherapy. However, the CD30 antigen releases the soluble ectodomain of CD30, which might obscure the targeted therapy. Therefore, the membrane epitope of CD30 (mCD30), left on the cancer cells, might be a prospective target for lymphoma treatment. The discovery of novel mCD30 monoclonal antibodies (mAbs) using phage technology yielded 59 potential human single-chain variable fragments (HuscFvs). Ten candidate HuscFv clones have been selected based on various methods, i.e., direct PCR, ELISA and western blot assays, and nucleotide sequencing techniques. Fortunately, only one potential HuscFv clone, clone #A4, was determined by the prediction of HuscFv-peptide molecular docking and the binding affinity test using isothermal titration calorimetry. Finally, we proved that the HuscFv #A4, which had a binding affinity (*K*_d_) of 421e-9 ± 2.76e^-6^ M, might be the novel mCD30 mAb. We generated chimeric antigen receptor-modified T lymphocytes using HuscFv #A4 as an antigen detection part (anti-mCD30-H4CART). The cytotoxicity assay of anti-mCD30-H4CART cells showed significant eradication of the CD30-expressing cell line, K562 (p = 0.0378). We found a novel mCD30 HuscFv using human phage technology. We systematically examined and proved that our HuscFv #A4 could specifically eradicate CD30-expressing cancers.

## Introduction

The standard treatment for Hodgkin’s lymphoma (HL) and anaplastic large cell lymphoma (ALCL) involves combining chemotherapeutic drugs with or without irradiation. Some of the affected patients, however, might have relapsed or refractory diseases [1]. These cancerous lymphoid cells (HL and ALCL cells) also have elevated CD30 antigens expression. A type I transmembrane glycoprotein belonging to the tumor necrosis factor receptor (TNFR) superfamily 8 (TNFRSF8), the human CD30 antigen. The membrane-anchored metalloproteinase TNF-α converting enzyme (TACE) has the ability to cleave the extracellular domain of the CD30 antigen within the juxtamembrane stalk, resulting in the release of the soluble CD30 (sCD30) [2, 3] Typically, CD30 is restricted on activated B, T, or null cells, and some CD4^+^ and CD8^+^ T cells populations generating cytokines of the Th2-type [4–7]. Depending on the cells involved and other costimuli, the interaction between CD30 and CD30 ligand has pleiotropic biological effects that range from causing apoptosis to promoting the survival of CD30^+^ cells [8–11]. The overexpression of CD30, however, is advantageous for anti-apoptotic mechanisms on malignant cells via a variety of signaling pathways sent by tumor necrosis factor receptor-associated factors (TRAF) [12]. The CD30 antigen is a well-known therapeutic marker for patients with HL and ALCL, the CD30-targeted immunotherapy [13–15].

Current antibody and cell-based therapies for HL and ALCL that target CD30 have been consistently improved. Nevertheless, earlier treatments modified restrictedly from the existing monoclonal antibodies (mAbs) derived from murine origin or chimeric modifications led to human anti-mouse Antibodies (HAMA) toxicities and eventually disappointing outcomes in clinical trials [16]. Previous studies examined whether some mAb binding domains, single-chain Fv fragments (scFvs), such as Ki-1, Ki-2, R4-4, Ber-H2, and HRS-3, recognized the sCD30 [17]. Certain scFvs, such as Ki-1, Ki-2, Ki-3, Ki-5, HeFi-1, and M44 mAbs, increase the shedding of the sCD30, and only a few scFvs, as Ki-4 and Ber-H2, inhibit the shedding of the sCD30 [2, 18]. Furthermore, because the sCD30 altered the biodistribution of these agents before they reached their tumor target site, it had the impact of neutralizing and reducing the activities of therapeutic CD30-targeting mAbs [18]. However, the production of mAbs or recombinant antibodies were not concerned about the specificity of the mAbs to target epitopes [19]. Additionally, a receptor chimera known as the chimeric antigen receptor (CAR), which combines a scFv component with a stimulating domain to provoke immune cells, has been developed. Recently, FDA-approved treatment of hematologic malignancies with infusions of CAR-modified T lymphocytes (CART cells) to patients harboring B-cell hematologic malignancies, i.e., acute leukemia, lymphomas, BCMA-expressing diseases, and multiple myeloma was launched worldwide [20].

One strategy to improve CD30-targeted immunotherapy is to explore novel membrane CD30 (mCD30)-specific epitopes as target epitopes due to no competition with sCD30 combined with the recent FDA-approved technique, CAR T cells [19, 21, 22]. In order to address the issue of the sCD30-neutralizing, reduce HAMA toxicities, and enhance CD30-targeted immunotherapy, this study aimed to discover human scFvs that recognize mCD30 epitopes (mCD30-scFvs). Then we would apply the identified mCD30-scFVs to a CAR construct, express the CAR on T lymphocytes to create anti-mCD30-CART cells, and test their cytotoxic functions. We would like to prove the concept that these anti-mCD30-CART cells would be an alternative method for CD30-expressing tumor treatment.

## Materials and methods

### 1. Phage bio-panning and clonal selection

A 96-well Pierce Streptavidin-coated high-capacity plate (Thermo Scientific, USA) was laminated with 2.5 μM of synthetic mCD30 peptide (47 amino acids; GenScript, USA) in 100 μL of Pierce protein-free (PBS) blocking buffer, pH 7.4 (Thermo Fisher Scientific, USA). The HuscFv phage library, kindly provided by Prof. Dr. Wanpen Chaikumpa (Center of Research Excellence on Therapeutic Proteins and Antibody Engineering, Department of Parasitology, Faculty of Medicine Siriraj Hospital, Mahidol University, Thailand), was added to the peptide-coated wells [23, 24]. After removing unbound phages, the log-phage *E*. *coli* HB2151 was permitted to infect for 10 minutes, then spread onto LB-A agar plates. Phagemid-transformed *E*. *coli* colonies on the plates were screened for scFv genes (*scFvs*) by direct colony PCR. The *scFvs*-positive *E*. *coli* clones were induced to express scFv proteins (ScFvs), and then soluble *E*. *coli* fractions containing ScFvs were collected [24]. An indirect ELISA assay was performed on the binding of ScFvs in the soluble *E*. *coli* fractions to the mCD30 peptide, while a blocking buffer was used as a negative control. The absorbance values were measured at 405 nm. The ScFvs containing *E*. *coli* clones that showed and OD_405nm_ signal above mean + 3SD of the background binding control (lysate of original *E*. *coli* HB2151, HB) were selected as bound phages. Besides, the bound ScFvs in the *E*. *coli* fractions were confirmed by western blot using an anti-E tag antibody (Abcam, USA) as the E-tagged-ScFv tracer. Phagemid DNAs from the selected *E*. *coli* clones, producing anti-mCD30 ScFvs, were subjected to nucleotide sequencing by the GeneArt Gene Synthesis (ThermoFisher Scientific, USA). The complementarity-determining regions (CDRs) and immunoglobulin framework regions (FRs) of all sequences were identified using the integrative database of germ-line variable genes (VBASE2, http://www.vbase2.org).

### 2. Computerized simulation

Amino acid sequences of the mCD30 peptide epitope and the ScFv candidate clones were subjected to homology modeling by iterative threading assembly refinement (I-TASSER) [25]. The I-TASSER predicted models were refined to improve the physical quality of the predicted 3D structure using high-resolution protein structure refinement, i.e., ModRefiner [26]. The 3D structure of the target peptide and the modeled ScFvs were subjected to protein-peptide docking (CABS-dock). All models were visualized using PyMol software (PyMol Molecular Graphics System, Version 2.5, Schrodinger, LLC).

### 3. ScFvs expression, purification, and binding properties

The *scFvs* of the *E*. *coli* clones were optimized and synthesized to conjugate with Flag-tag DNA sequence at the C-terminal before subcloning into the pET24-b(+) expression vector under the T7 promoter. Large-scale production of soluble Flag-tagged-ScFvs was performed by transforming the recombinant pET24-b(+) *scFv* plasmids into the *E*. *coli* SHuffle T7 express (New England Biolabs Inc., USA). The *scFv*-positive *E*. *coli* clones were grown in LB broth containing 50 μg/mL of kanamycin (LB-K) at 37°C before inducing with isopropylthio-β-galactoside (IPTG) and shaking at 30°C for 16 hours. After extracting the bacteria cells with a sonicator on ice, the soluble Flag-tagged-ScFvs were collected and purified from the *E*. *coli* fractions using anti-DYKDDDDK affinity resin beads (Pierce, Thermo Scientific, USA). The purified scFv was used to evaluate the binding affinity by Isothermal Titration Calorimetry (ITC). The purified scFvs #AK and #GD2, specific to CD30 and GD2 antigens, respectively, were used as controls [27, 28]. For changing the buffer to HBS-P buffer pH 7.4, Amicon Ultra-15 Centrifugal Filter Units (Merck Millipore, Darmstadt, Germany) were used. Pierce^™^ BCA Protein Assay Kit (Thermo Scientific, USA), SDS-PAGE, and western blot using the anti-FLAG M2 antibody (Sigma-Aldrich, USA) were used to determine protein quantity and quality. The measurement of heat generated from the binding reaction in the ITC machine was analyzed and converted to the thermodynamic parameters; Gibbs free energy (ΔG), entropy (ΔS), and enthalpy (ΔH), including binding affinity (*K*_d_) and stoichiometry (n).

### 4. Cell lines

K562 cells (Human Caucasian chronic myelogenous leukemia) and HEK293T cells (Human embryonic kidney cell line) were grown in the RPMI 1640 media (Cytiva *HyClone*^™^, Fisher Scientific, UK) and DMEM (Dulbecco’s Modified Eagle’s Medium) (Cytiva *HyClone*^™^, UK), respectively, supplemented with 10% heat-inactivated fetal bovine serum (FBS) (Hyclone, GE Healthcare, USA) and 100 units/mL of penicillin/streptomycin (Gibco, USA) at 37°C in a 5% CO_2_ atmosphere. SupB15 cells (Human Lymphoblastic Leukemia) were cultured in Iscove’s modified Dulbecco’s medium with 4 mM L-glutamine adjusted to contain 1.5 g/L sodium bicarbonate and supplemented with 0.05 mM 2-mercaptoethanol and 20% FBS.

### 5. Generation of anti-mCD30 CAR lentiviruses

The chimeric DNA sequence of CAR was designed and optimized for humanized codon usage in the order of the extracellular domain (EC) of CD8, the transmembrane domain (TM) of CD8, and the cytosol CD3-ζ including GFP as a marker by GenScript. After humanized codon-optimization, the selected *scFvs* were inserted into the CAR construct and subcloned into the pSIN-EF2-LIN28-Pur expression vector under the EF-1α promoter. The second generation of the lentiviral expression cassettes was used to produce ScFv-CAR lentiviruses by using HEK293T as packaging cells [28]. The CAR virus titer was checked using the Lenti-X qRT-PCR Titration Kit (Takara Bio, USA). The GFP fluorescence was observed in the transduced T cells using an inverted fluorescent microscope at 20x.

### 6. T cells isolation and transduction

Peripheral blood mononuclear cells (PBMC) at the buffy coat were isolated from healthy donors, resuspended in the completed RPMI 1640 media, and cultured in the anti-Hu CD3 clone OKT3 (eBioscience, Invitrogen, USA)-coated plate at 2 x 10^6^ cells/mL, adding anti-Hu CD28 clone 28.2 (eBioscience, USA). The next day, 200 U/mL of human interleukin 2 (h-IL-2) (Shenandoah Biotechnology, USA) was added to each well. The transduction was performed on the third day [28]. After culturing cells for seven days, the immunophenotyping, percentage of transduction efficiency, and cytotoxicity assays were performed.

The study was approved by the Ethical Clearance Committee on Human Rights Related to Research Involving Human Subjects, Faculty of Medicine Ramathibodi Hospital, Mahidol University (MURA2020/694). The written consents were obtained from all the participants involved in this study.

### 7. Immunophenotyping of transduced T cells

The immunophenotype of T cells was performed by staining marker antigens on the surface of the cells with Ms mAb to CD3 [UCHT1] PerCP (Abcam, USA), Ms mAb to CD4 [B-A1] FITC (Abcam, USA), Ms mAb to CD8 [MEM-31] APC (Abcam, USA), Ms mAb to CD45RA [MEM-56] PE/Cy7 (Abcam, USA), and Ms mAb to CD62L [LT-TD180] PE (Abcam, USA) before detecting the expression of surface markers by flow cytometry (BD Biosciences, USA).

### 8. Transduction efficiency of transduced T cells

Transduction efficiency was checked by amplifying a CAR-DNA fragment in the genomic DNA of transduced T cells with real-time PCR using primer probes and SsoAdvanced^™^ Universal Probes Supermix (Bio-Rad Laboratories, USA). The standard curve of viral copy number and Cq values was generated. The Cq values were converted to the viral copy number by calculating from the standard curve.

### 9. Cytotoxicity assay

CD30+ cell lines, K562 cell lines, and CD30- cell line, SupB15, were stained with 1μM of CellTrace^™^ Violet cell proliferation kit (Life Technologies Corporation, ThermoFisher Scientific, USA) and co-cultured individually with engineered- and mock T cells at effector-to-target ratios (E: T) of 1:1 and 40:1. After 24 hours of culture, the cell pellet was stained with 7-amino-actinomycin D (7AAD) (eBioscience, USA) to detect the proportion of dead target cells and analyzed by Navios Flow Cytometry (Beckman Coulter, USA) and FlowJo software (version 10, TreeStar, USA). The specific lysis was calculated as follows;

%SpecificLysis=(sampledeath-spontaneousdeath)/(100-spontaneousdeath)x100

## Results

### Phage bio-panning and clonal selection

**Fig 1:**
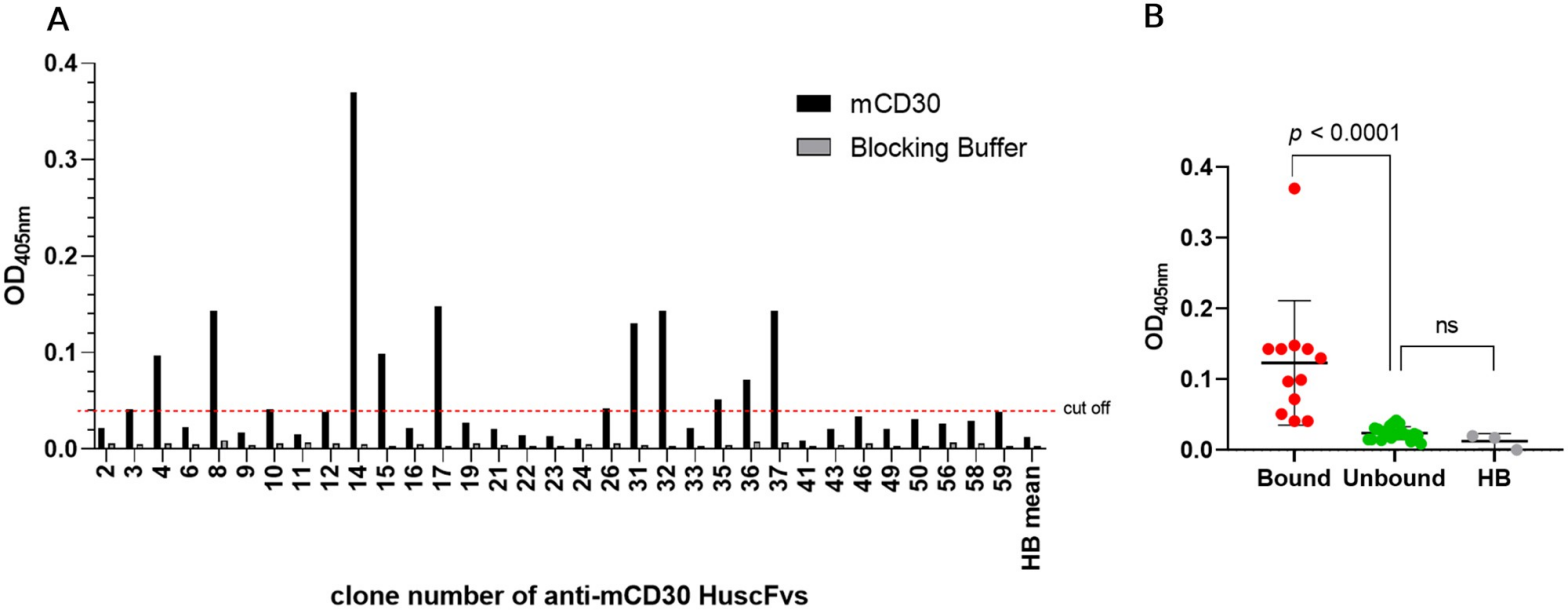
Clonal selection of mCD30-bound ScFvs. **(A)** The screening of *E*. *coli* clones that produced mCD30-bound ScFvs by indirect ELISA used the mCD30 peptide as a peptide-coated plate and the blocking buffer as a non-coated plate (negative control) in the side-by-side wells. Bound ScFvs were selected from the OD_405nm_ signal above the mean + 3 SD of the background binding control (lysate of original *E*. *coli* HB2151; HB) (cut off = 0.03875). Lysates of 12 *scFv*-positive *E*. *coli* clones (#A3, A4, A8, A10, A14, A15, A17, A31, A32, A35, A36, and A37) produced soluble ScFvs bound to the mCD30 peptide. **(B)** The statistical significance between the bound, unbound ScFvs groups and the HB control was analyzed by one-way ANOVA and Tukey’s post hoc test.

By phage bio-panning using the mCD30 peptide as an antigen, 59 colonies of *E*. *coli* HB2151 transfected with mCD30-bound phages were selected from the human ScFv-phage display library. Thirty-three clones carried *scFvs* with PCR amplicons at ~ 1,000 bp (S1 Fig). Lysates of 12 *scFv*-positive *E*. *coli* clones produced soluble ScFvs bound to mCD30 (Fig 1A, S2 Fig) using a blocking buffer as a negative control. The statistical analysis between the bound and unbound ScFvs was determined using a one-way ANOVA and Tukeyʼs post hoc test (Fig 1B). From nucleotide sequencing, ten clones (#A3, A4, A8, A10, A14, A15, A17, A31, A32, and A36) contained complete scFv sequences of a variable heavy chain (VH), linker (GGGGS)_3_, and variable light chain (VL). These clones were classified into eight different types based on the deduced amino-acid sequences and numbering of the Kabat and Chothia scheme [29]; type 1 (IGHV3 family and IGKV2: A3), type 2 (IGHV5 family and IGKV3: A4), type 3 (IGHV1 family and IGKV1: A8, A31), type 4 (IGHV3 family and IGKV1: A10), type 5 (IGHV3 family and IGKV1: A14, A15), type 6 (IGHV5 family and IGKV1: A17), type 7 (IGHV1 family and IGKV3: A32), type 8 (IGHV4 family and IGKV1: A36).

### Computerized simulation

**Fig 2:**
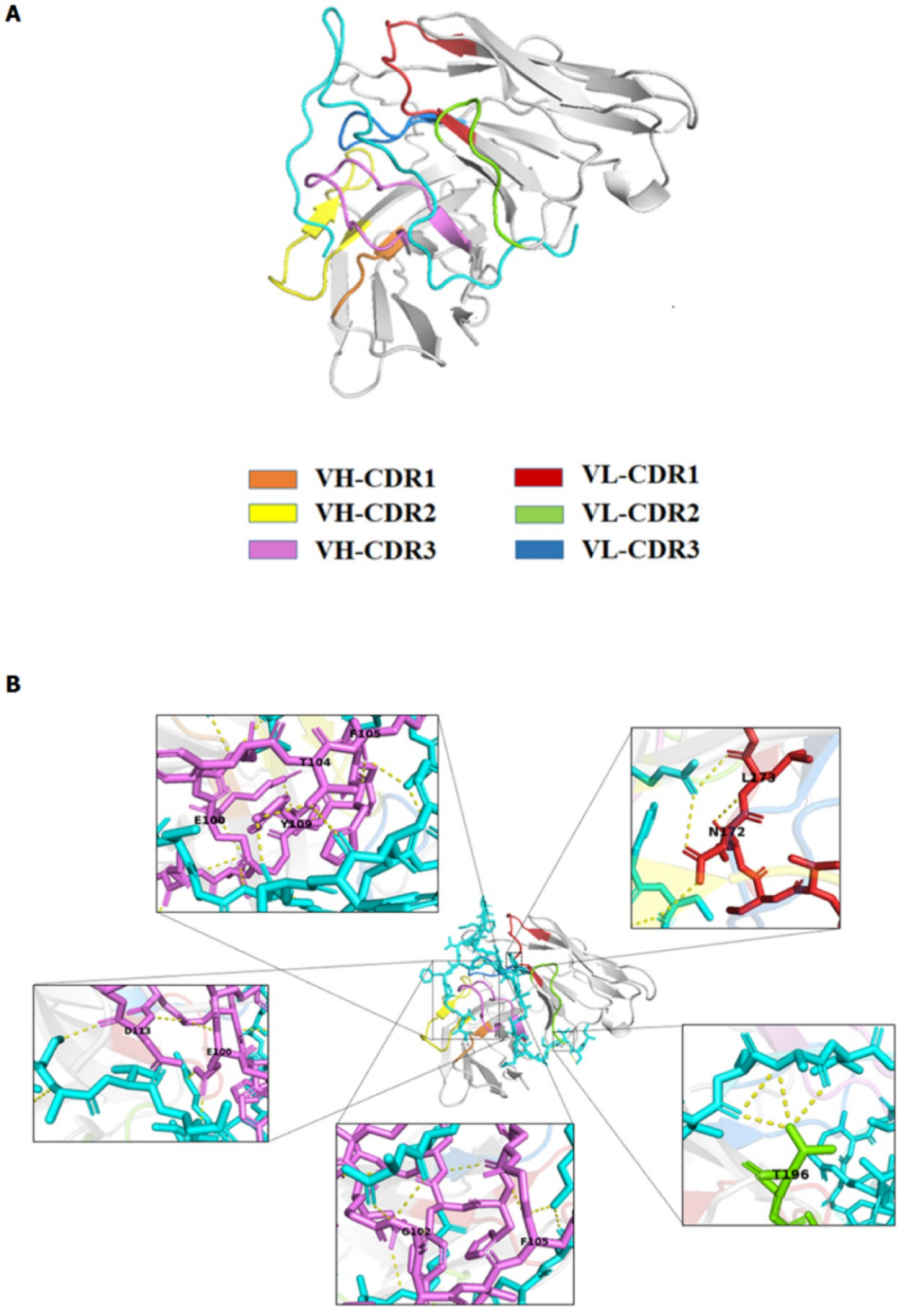
Computerized simulation of the 3D structure of mCD30-ScFv complex and presumptive contact interfaces. **(A)** The complexing of the mCD30 peptide (cyan) and the ScFv (grey) of *E*. *coli* clone #A4 after simulation from the molecular docking showed the contact interaction at the complementarity-determining regions (CDRs) binding sites of the ScFv. **(B)** Contact interfaces with hydrogen bonding between mCD30 and CDRs of ScFv#A4 at G102, T104, F105, Y109, D113, N172, L173, T196, and a salt bridge at E100).

The 3D structure of individual models of the ScFvs and the mCD30, translated from amino acid sequences was subjected to intermolecular docking to predict the presumptive residues of the contact interface. Of the candidates, the ScFv of *E*. *coli* #A4 as showed the contact interaction at the CDR binding sites, as shown in Fig 2A, and the presumptive binding site of each clone, shown in Fig 2B, respectively. The docking *in silico* showed that the CDRs of the ScFv#A4 formed hydrogen bonds with the mCD30 peptide epitope at G102, T104, F105, Y109, D113, N172, L173, T196, and developed a salt bridge at E100. Whereas the rest candidates showed non-specific binding, one of the criteria we used to exclude non-specific clones. (the representative candidate in S3 Fig).

### ScFvs expression, purification, and binding properties

**Fig 3:**
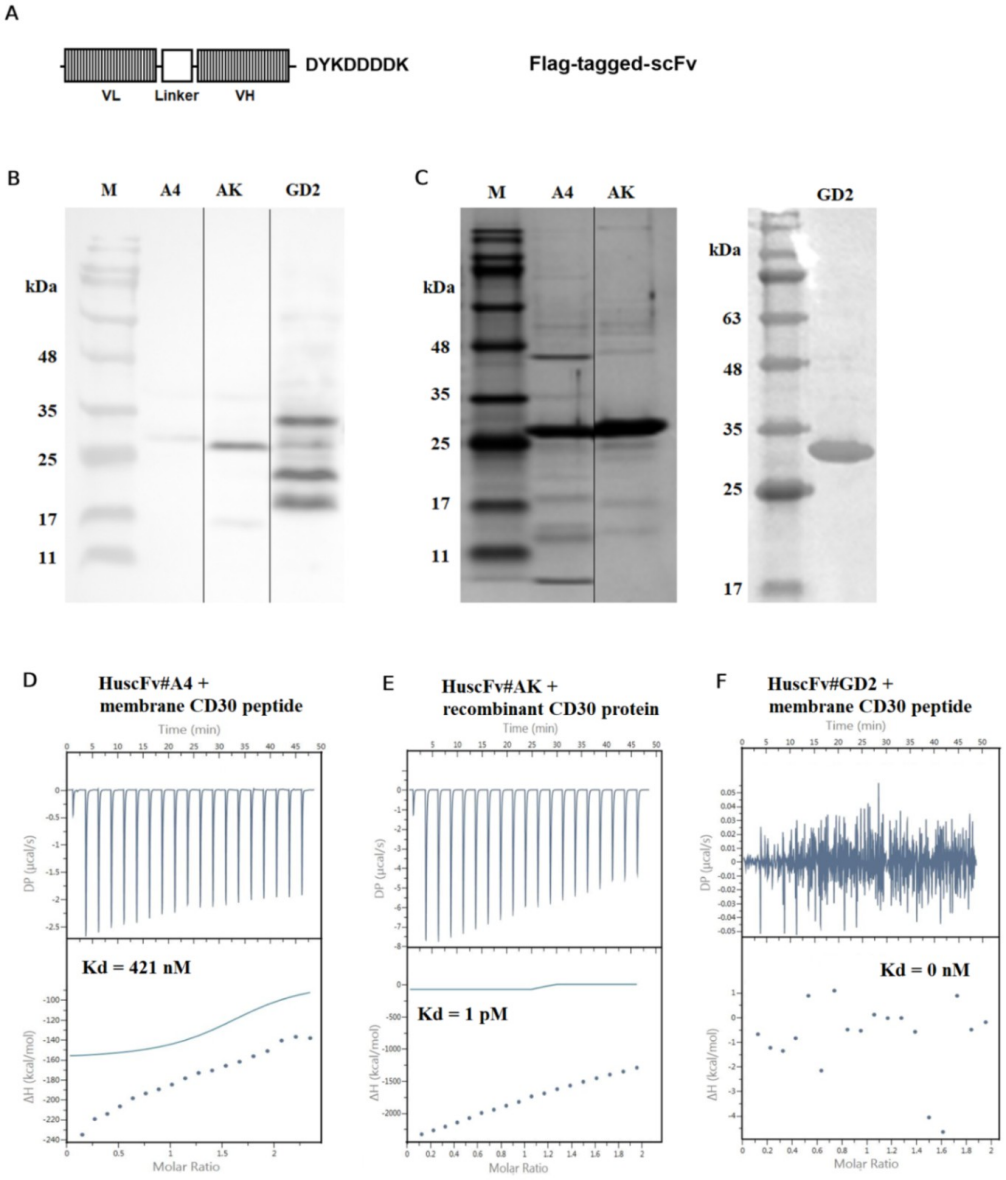
Expression, purification, and binding properties of ScFvs. **(A)** Schematic representation of Flag-tagged-ScFv in the pET24-b(+) expression vector under the T7 promoter. **(B)** The expression levels of soluble Flag-tagged-ScFv #A4 (28.216 kDa), #AK (positive control: 27.975 kDa), and #GD2 (negative control: 33.675 kDa) presented in the *E*. *coli* fractions detected by western blot. **(C)** The 5 μg purified Flag-tagged-ScFv#A4, #AK, and #GD2 were resolved on 12% SDS-PAGE. **(D-F)** Binding affinity between ScFv#A4, #AK, and #GD2 and the targets by Isothermal Titration Calorimetry (ITC). Thermograms were recorded at 25°C. ScFv#A4 and ScFv#AK (positive control) revealed an exothermic association with *K*_d_ of 421 nM and 1 pM, respectively. In contrast, ScFv#GD2 (negative control) showed no binding.

The *scFv* of *E*. *coli* #A4 was optimized and synthesized to conjugate with the Flag-tag DNA sequence at the C-terminal before subcloning into the pET24-b(+) expression vector (Fig 3A). The recombinant plasmid was transformed into the *E*. *coli* SHuffle T7 express to produce large amounts of soluble ScFv under IPTG inducer conditions. The expression level of Flag-tagged-ScFv was detected by western blot (Fig 3B). The Flag-tagged-ScFvs were purified out of *E*. *coli* fractions, and the purity was confirmed by the SDS-PAGE technique (Fig 3C). The binding affinity (*K*_d_) of the purified ScFvs#A4, AK, and GD2 were determined using Isothermal Titration Calorimetry (ITC). Titrations of mCD30 and recombinant CD30 (rCD30) peptides into the ScFv#A4 and the ScFv#AK (positive control) revealed an exothermic association with *K*_d_ of 421 nM and 1 pM, respectively (Fig 3D and 3E, respectively, and S1 Table). The association of the ScFv#GD2 (negative control) showed no binding (Fig 3F and S1 Table).

### Generation, transduction, and cytotoxicity of anti-mCD30 CAR T cells

**Fig 4:**
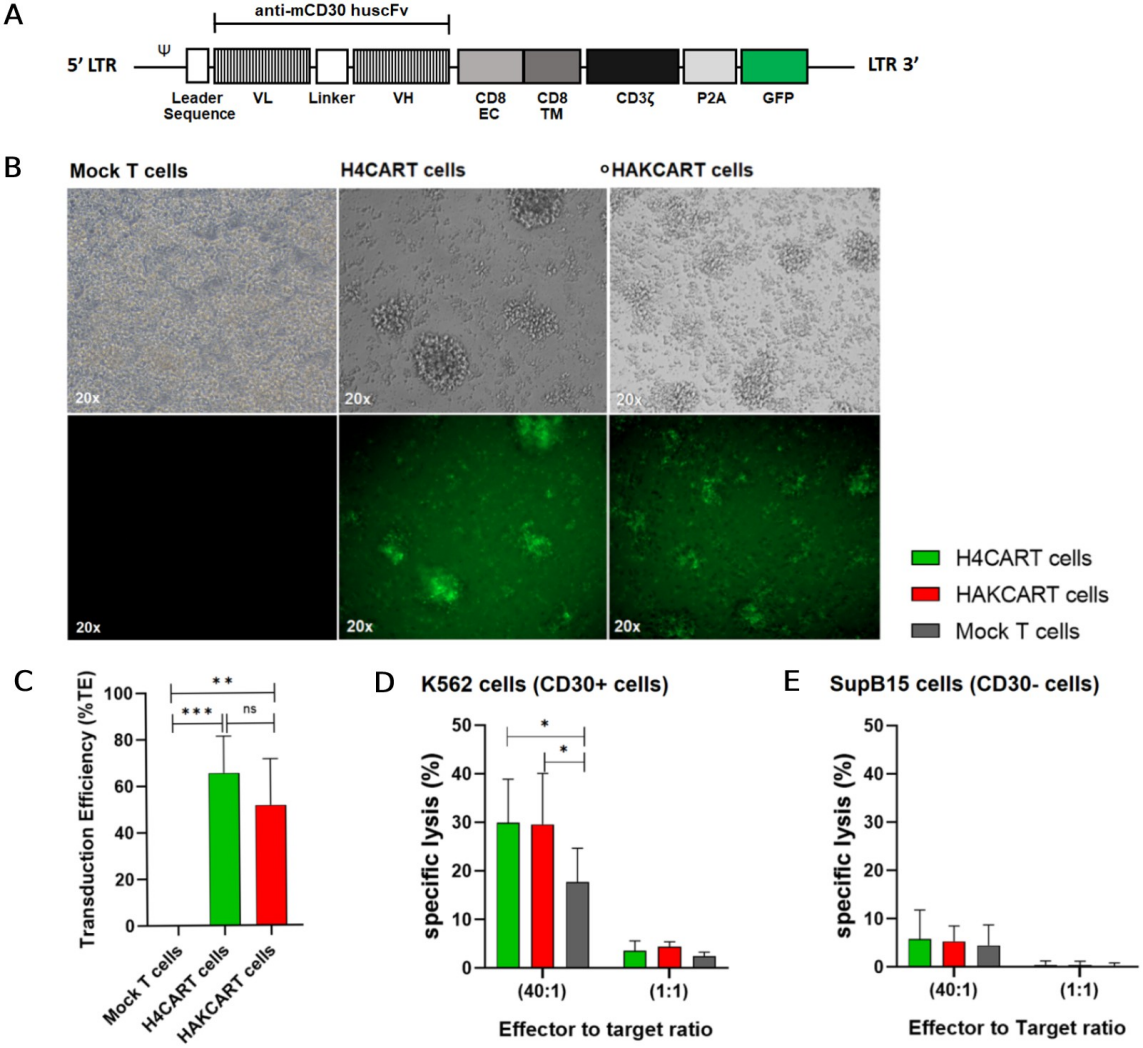
Transduction, characteristic, and cytotoxic assays of anti-mCD30 CAR T cells. **(A)** Schematic representation of the anti-mCD30 *scFv*-CAR lentiviral vector. **(B)** GFP of transduced T cells with *scFv*#A4-CAR lentivirus, H4CART, and *scFv*#AK-CAR lentivirus; HAKCART was observed under an inverted microscope at 20x; bright field (upper panel) and fluorescent field (lower panel). **(C)** The transduction efficiencies (%TE) of engineered T cells were significantly different from mock T cells (p = 0.0004 and p = 0.002, n = 4, one-way ANOVA (Tukey’s multiple comparisons test)), but there was no significant difference between both populations of engineered T cells (p = 0.4089, one-way ANOVA (Tukey’s multiple comparisons test)). **(D)** At an E:T ratio of 40:1, the cytotoxicity of H4CART and HAKCART cells on K562 cells was significantly higher than mock T cells (p = 0.0378 and p = 0.0439, respectively; n = 4, two-way ANOVA (Tukey’s multiple comparisons test)). **(E)** In SupB15 cells, the specific lysis of the engineered T cells was not significantly different from mock T cells at all E:T ratios.

Generation of anti-mCD30 CAR, the humanized, codon-optimized *scFv*-CAR construct, was subcloned into the pSIN-EF2-LIN28-Pur lentiviral expression vector (Fig 4A). The activated T cells were transduced with individual CAR lentiviruses at an MOI of 100, and GFP fluorescence was observed in H4CART (*scFv*#A4-CAR) and HAKCART (*scFv*#AK-CAR) cells (Fig 4B).

The percentage of transduction efficiency (%TE) of engineered T cells was calculated as shown in S4 Fig. The result showed that H4CART cells and HAKCART cells were significantly different from mock T cells (p = 0.0004 and p = 0.002, respectively, n = 4, one-way ANOVA (Tukey’s multiple comparisons test)), but there was no significant difference between both populations of engineered T cells (p = 0.4089, one-way ANOVA) (Fig 4C). The characteristics of each group of T cells are shown in S5 Fig.

Before cytotoxic testing, CD30 expression on the cell surface of K562 and SupB15 cells was detected (S6 Fig). Due to the negative expression of CD30 on SupB15 cells, it was chosen as a CD30 negative control cell in the subsequent experiment.

In K562 cells, the specific lysis (%) of H4CART and HAKCART cells was significantly higher than mock T cells at an E:T ratio of 40:1 (p = 0.0378 and p = 0.0439, respectively; n = 4, two-way ANOVA (Tukey’s multiple comparisons test)) (Fig 4D). In SupB15 cells, the specific lysis of all engineered T cells was not entirely different from mock T cells at all E:T ratios (Fig 4E).

## Discussion

Overcoming the drawbacks of previous CD30-mAbs, sCD30 neutralization, and HAMA toxicities, we discovered a novel ScFv specific to mCD30 epitopes by biopanned with the available human ScFv phage library and confirmed by various methods. Moreover, this ScFv showed intermolecular docking of their 3D structures with that of the mCD30 peptide at their CDR binding sites, as confirmed by the binding assay using the ITC. Application of this newly identified ScFv into the CAR construct would allow us to demonstrate its transduction efficiency, immunophenotype, and cytotoxicity. Fortunately, we could prove that this new human ScFv, specific to the mCD30 epitope, could eradicate CD30+ hematologic malignant cell lines in vitro, which may enlighten CD30-targeted immunotherapy.

The PCR techniques defined the biopanning-screened positive phage clones by selecting the potential mCD30-specific ScFvs, derived from approximately 1,000 base pairs of *scFvs*. The *scFv*-positive *E*. *coli* clones, which had incomplete ScFvs of approximately 17 kDa, bound to the mCD30 peptides, might result from the incomplete *scFv* DNA sequence itself or the dissociation of the ribosome from the *scFv* mRNA in the translation of protein synthesis [30]. The latter might be from the rare codon of *scFv* messenger RNAs (mRNA), which transfer RNA (tRNA) could bind to amino acids, leading to no elongation of protein synthesis, or might be because of impaired upstream Open Reading Frames (ORFs), resulting in damaging the translation of the main ORF [31].

The 3D structures of the intermolecular dockings between the individual bound ScFv and the mCD30 peptide were visualized to investigate the specific CDR binding sites of each ScFv clone. Our criteria for selection of the optimal models of the ScFv-mCD30 binding complex were 1) the lowest Gibbs free energy generation, which happened in spontaneous binding, and 2) a close distance (<3.0 Å) of the contact interface with the hydrogen bonds between the residues of either CDR-H or CDR-L in the ScFvs and mCD30 peptide [32]. From the visualization, ScFv clone A4 showed the characteristics as the criteria. Although the others might have higher binding results from the indirect ELISA test, they might have non-specific interactions because the free CDR binding sites could interact with other molecules, leading to non-specific binding.

Production of large-scale potential ScFv used the artificial mutant bacteria *E*. *coli* strain SHuffle T7 since this stain could produce a high yield of soluble and functional ScFv in the cytoplasm with the proper folding and without the requirement of refolding [33]. Additionally, the design of a potential ScFv conjugated with the DYKDDDDK tag assisted in maintaining the stability and solubility of the ScFv in the expression process. Furthermore, this tag was a marker for purifying the soluble ScFv-tagged with anti-DYKDDDDK affinity tag resin beads. The potential of purified ScFv revealed strong binding affinity (*K*_d_) based on favorable enthalpy and unfavorable entropy [34].

The cytotoxic result in the CD30+ hematologic malignant cell lines, K562 cells, had a similar pattern to the positive control. The mCD30-H4CART cells statistically significantly eradicated specific targets. However, the positive control should have a higher specific lysis due to its stronger binding affinity (*K*_d_). This finding might be due to the humanized clone of the positive control from its murine monoclonal antibody retaining the determinant for epitope-specificity and reducing its antigenicity [27]. Therefore, the humanized clone still recognizes the same determinant on the sCD30, which might diminish its efficiency [18]. Furthermore, the lower %TE of the positive control might affect their cytotoxic lysis on targets.

The specific lysis of both engineered T cells on the SupB15 cells (CD30-) was not significantly different from mock T cells at both E:T ratios. The cytotoxicity was very low due to no binding between the CAR and its target antigen, leading to no immunological synapse (IS) formation, which is the initial step that induces apoptosis and releases cytokines [35].

## Conclusion

In conclusion, we produced the anti-mCD30-CART cells, H4CART cells, which could activate the specific killing effects by using the CAR without the requirement of the TCR function. In addition, this novel mCD30 mAb, ScFv#A4, recognized the membrane CD30 epitope, not the extracellular domain of CD30 epitopes, which might lead to an increased function of CD30-targeted immunotherapy, including engineered T cells. Although the efficacy of H4CART cells was moderate, we might incorporate other means specific to PD-L1 to improve our engineered T cells’ specificity and cytotoxic function. Finally, we proved that these H4CART cells, against the mCD30 antigen, would be another method that might modify CD30 expressing tumor treatment.

## Supporting information

S1 Table The thermodynamic parameters of the purified protein binding determined by the isothermal titration calorimetry (ITC). Titrations of membrane CD30 (mCD30) and recombinant CD30 (rCD30) peptides into the ScFv#A4 and the ScFv#AK (positive control) revealed an exothermic association based on favorable enthalpy and unfavorable entropy with a binding affinity (*K*_d_) of 421 nM and 1 pM, respectively. The stoichiometry (N) of the ScFv#A4 and the ScFv#AK showed 1.66 ± 1.93 and 1.10 ± 2.87, respectively. (n = 3).
(DOCX)

S1 Fig The screening of membrane CD30 (mCD30)-bound phages. The screening of *E*. *coli* HB2151 clones that were transfected with mCD30-bound phages used direct PCR. The size of PCR amplicons was separated by gel electrophoresis. The sizes of a human single-chain Fv (*huscFv*), a human single-domain (*husdFv*) fragment, and an empty vector are about 1,000 bp, 500–700 bp, and 250 bp, respectively. Thirty-three clones, as indicated with red arrows, were carrying *huscFv* of mCD30-bound phages, which were clone #A2, A3, A4, A6, A8, A9, A10, A11, A12, A14, A15, A16, A17, A19, A21, A22, A23, A24, A26, A31, A32, A33, A35, A36, A37, A41, A43, A46, A49, A50, A56, A58, and A59.
(TIF)

S2 Fig The confirmation of soluble-bound human single-chain Fv proteins (HuscFvs) expression in *E*. *coli* fraction. The expression of soluble bound HuscFvs in *E*. *coli* fraction extracted from bacteria cells was confirmed by SDS-PAGE (upper panels) and western blot (lower panels) using an anti-E tag antibody as the E-tagged-HuscFv tracer. The soluble bound-HuscFvs (approximately 35 kDa) from human single-chain Fv (*huscfv)*-positive *E*. *coli* clone #A3, A4, A8, A10, A14, A15, A17, A31, A32, A35, A36, and A37 were detected.
(TIF)

S3 Fig The representative candidate of non-specific binding scFv. The intermolecular docking displayed the interactions between the representative candidate of a non-specific binding scFv clone and membrane CD30 peptide (cyan), which mainly occurred at the framework regions (grey) of the scFv structure, not the CDR binding sites. This scFv molecule, as a result, had free CDR binding sites to bind to other molecules, resulting in non-specific binding.
(TIF)

S4 FigThe calculation of transduction efficiency (%TE) by RT-PCR.The standard curve was performed using 50 ng of gDNA, which was referred to as cell number 7575.5 cells, plus various amounts of amplicon in each copy number starting from 10^3^ to 10^9^ copies. By which the amount of amplicon in each copy number was calculated from the formula below.

Numberofcopies=xng*6.0221*10^23molecules/moleN*660g/mole*10^9ng/g

x: the amount of amplicon (ng)N: the length of the dsDNA ampliconFifty ng of gDNA samples were used to amplify with RT-PCR. The starting quantity (SQ) from the amplification curve was used as a copy number of the sample to calculate the percentage of transduction efficiency (%TE), as shown in the formula below.%TE = copy number/cell number * 100Primer forward sequence: CTGGCAGGAACATGTGGCGTPrimer reverse sequence: CGTGGCTTGCCTCCCATCTCProbe: GCCGCTCCGCCGACGCACCA50 ng of gDNA = 7575.5 cellsAmplification curves and standard curves (n = 4).(TIF)

S5 Fig Immunophenotype of the engineered T cells detected by flow cytometry. Each group of T cells consisted of almost 100% CD3+ T cells, divided into CD8+ T cells and CD4+ T cells. The proportion of CD8+ T cells was higher than that of CD4+ T cells. Each population was classified into four subpopulations: naïve T cells (CD62L+, CD45RA+), effector T cells (CD62L-, CD45RA+), effector memory T cells (CD62L-, CD45RA-), and central memory T cells (CD62L+, CD45RA-). Effector memory and central memory T cells in CD4+ and CD8+ T cells showed higher levels than naïve and effector T cells.
(TIF)

S6 Fig The detection of CD30 expression. The CD30 antigen on the cell surface of healthy target cell lines (K562 and SupB15) was stained with PE anti-human CD30 antibody, and the expression was detected by flow cytometry. CD30 expresses at a consistently high level on the cell surface of K562 cells but not of SupB15 cells. The black-shaded histogram represented the unstain, and the blue showed CD30 staining.
(TIF)
