# Supplementary material for: A novel anti-membrane CD30 single-chain variable fragment discovered from the human phage library: A potential targeted immunotherapy
Source: PLoS One. 2023 Apr 20;18(4):e0284708. doi: 10.1371/journal.pone.0284708 (PMC10118152; doi:10.1371/journal.pone.0284708)
Supplement: S1 Table — Titrations of membrane CD30 (mCD30) and recombinant CD30 (rCD30) peptides into the ScFv#A4 and the ScFv#AK (positive control) revealed an exothermic association based on favorable enthalpy and unfavorable entropy with a binding affinity (Kd) of 421 nM and 1 pM, respectively. The stoichiometry (N) of the ScFv#A4 and the ScFv#AK showed 1.66 ± 1.93 and 1.10 ± 2.87, respectively. (n = 3). (DOCX) [file pone.0284708.s001.docx]

**S1 Table. The thermodynamic parameters of the purified protein binding determined by the isothermal titration calorimetry (ITC)** Titrations of membrane CD30 (mCD30) and recombinant CD30 (rCD30) peptides into the ScFv#A4 and the ScFv#AK (positive control) revealed an exothermic association based on favorable enthalpy and unfavorable entropy with a binding affinity (*K*_d_) of 421 nM and 1 pM, respectively. The stoichiometry (N) of the ScFv#A4 and the ScFv#AK showed 1.66 ± 1.93 and 1.10 ± 2.87, respectively. (n = 3)

|  | **ScFv#A4** | **ScFv#AK** |
| --- | --- | --- |
| ***K*_d_ (nM)** | 421 ± 2760 | 0.001 ± 279 |
| **∆H (kcal/mol)** | -80.0 ± 178 | -80.0 ± 1120 |
| **T∆S** | 71.3 | 63.6 |
| **N** | 1.66 ± 1.93 | 1.10 ± 2.87 |

Means ± SD are shown; N is the stoichiometry of binding
