# Supplementary figures and images for: A novel anti-membrane CD30 single-chain variable fragment discovered from the human phage library: A potential targeted immunotherapy
Source: PLoS One. 2023 Apr 20;18(4):e0284708. doi: 10.1371/journal.pone.0284708 (PMC10118152; doi:10.1371/journal.pone.0284708)

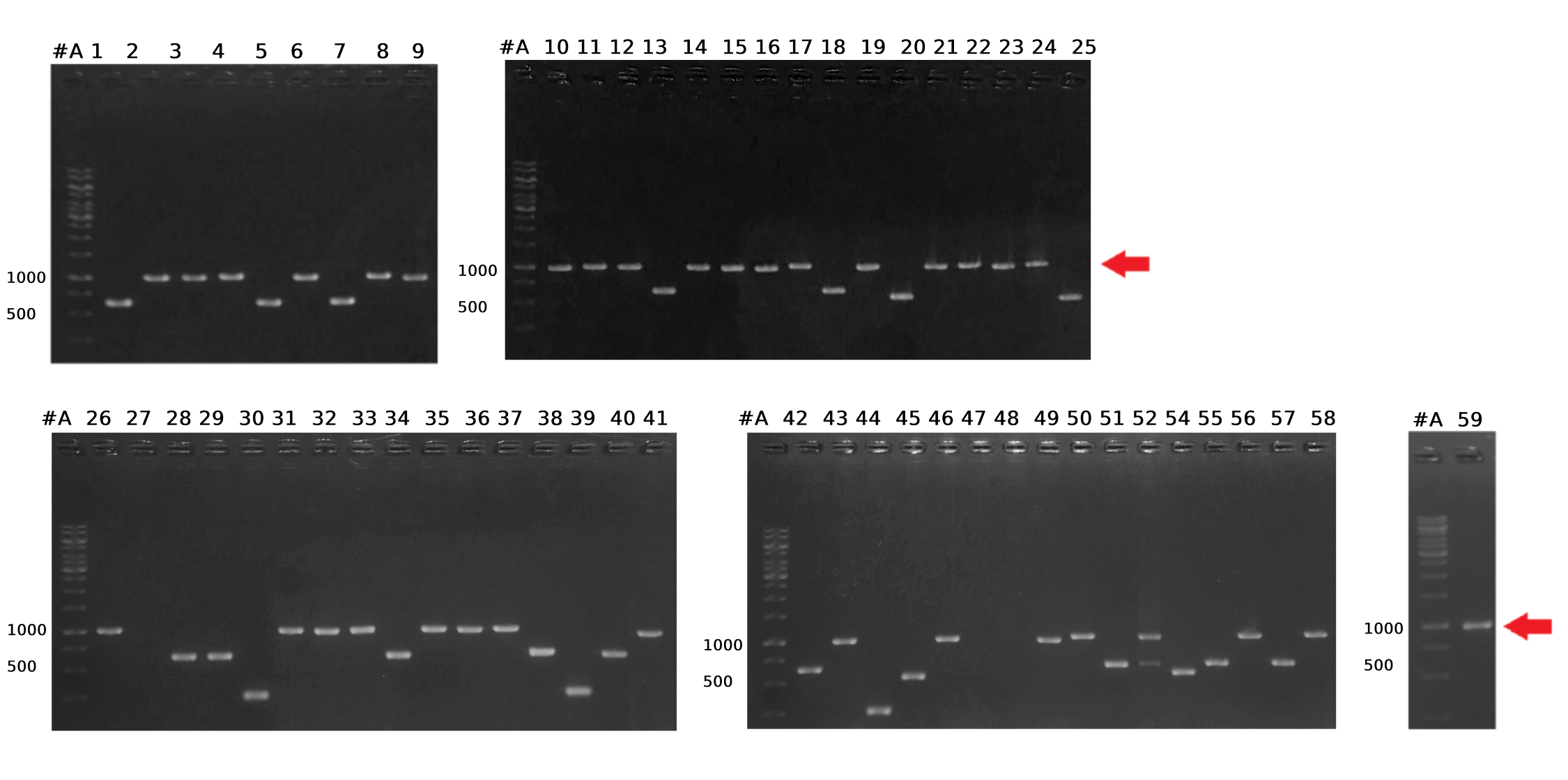

Supplement: S1 Fig — The screening of E. coli HB2151 clones that were transfected with mCD30-bound phages used direct PCR. The size of PCR amplicons was separated by gel electrophoresis. The sizes of a human single-chain Fv (huscFv), a human single-domain (husdFv) fragment, and an empty vector are about 1,000 bp, 500–700 bp, and 250 bp, respectively. Thirty-three clones, as indicated with red arrows, were carrying huscFv of mCD30-bound phages, which were clone #A2, A3, A4, A6, A8, A9, A10, A11, A12, A14, A15, A16, A17, A19, A21, A22, A23, A24, A26, A31, A32, A33, A35, A36, A37, A41, A43, A46, A49, A50, A56, A58, and A59. (TIF) [file pone.0284708.s002.tif]

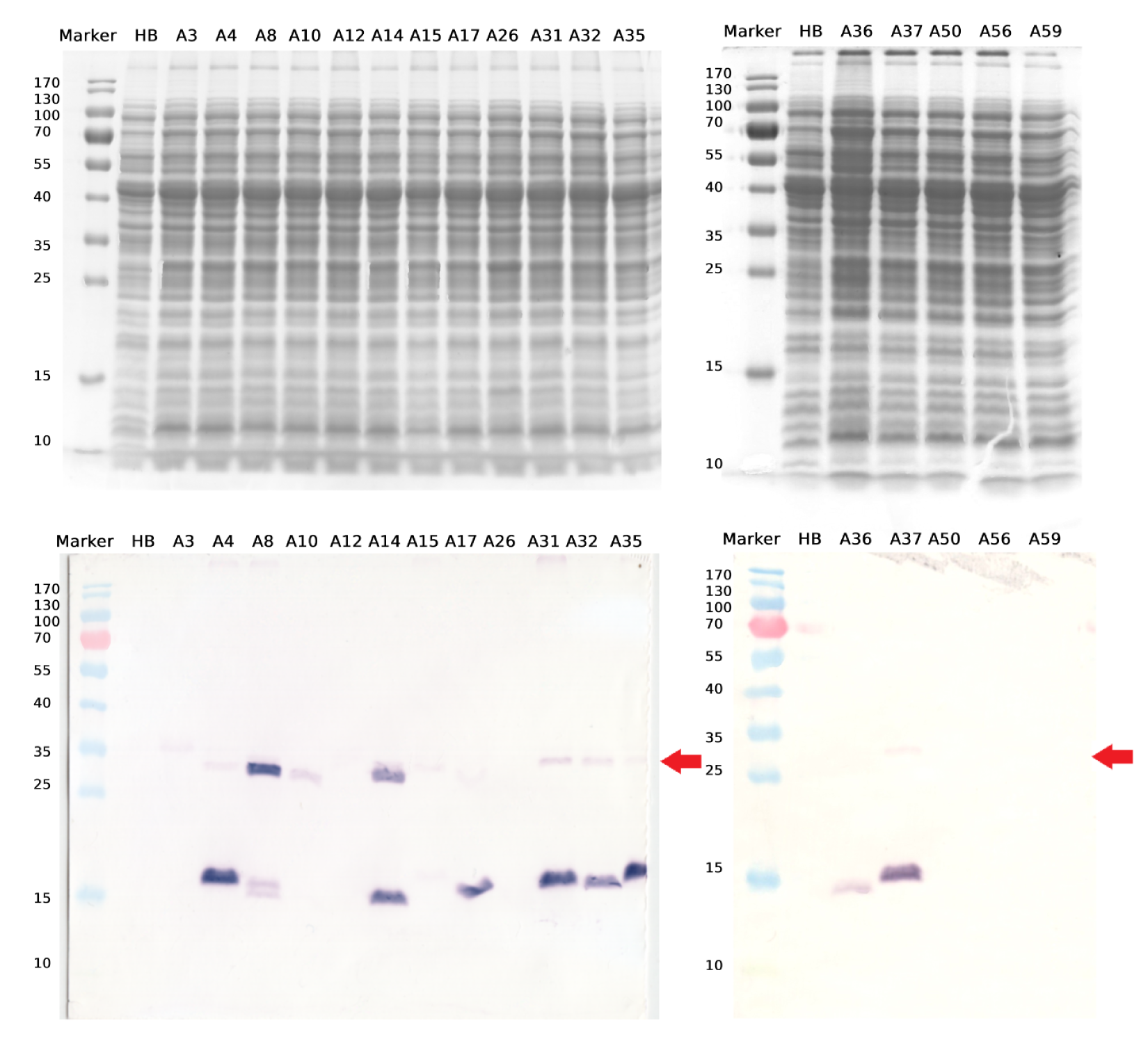

Supplement: S2 Fig — The expression of soluble bound HuscFvs in E. coli fraction extracted from bacteria cells was confirmed by SDS-PAGE (upper panels) and western blot (lower panels) using an anti-E tag antibody as the E-tagged-HuscFv tracer. The soluble bound-HuscFvs (approximately 35 kDa) from human single-chain Fv (huscfv)-positive E. coli clone #A3, A4, A8, A10, A14, A15, A17, A31, A32, A35, A36, and A37 were detected. (TIF) [file pone.0284708.s003.tif]

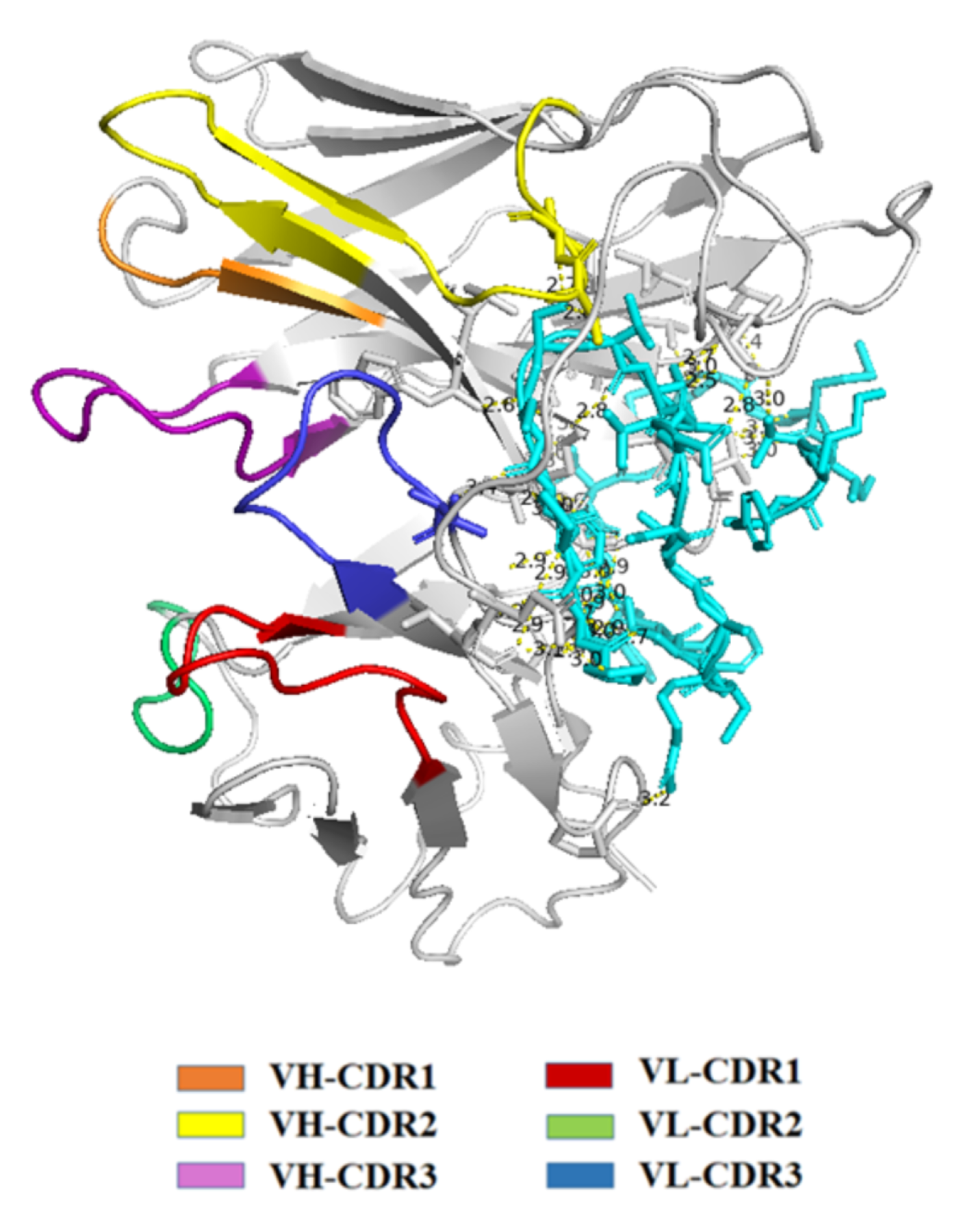

Supplement: S3 Fig — The intermolecular docking displayed the interactions between the representative candidate of a non-specific binding scFv clone and membrane CD30 peptide (cyan), which mainly occurred at the framework regions (grey) of the scFv structure, not the CDR binding sites. This scFv molecule, as a result, had free CDR binding sites to bind to other molecules, resulting in non-specific binding. (TIF) [file pone.0284708.s004.tif]

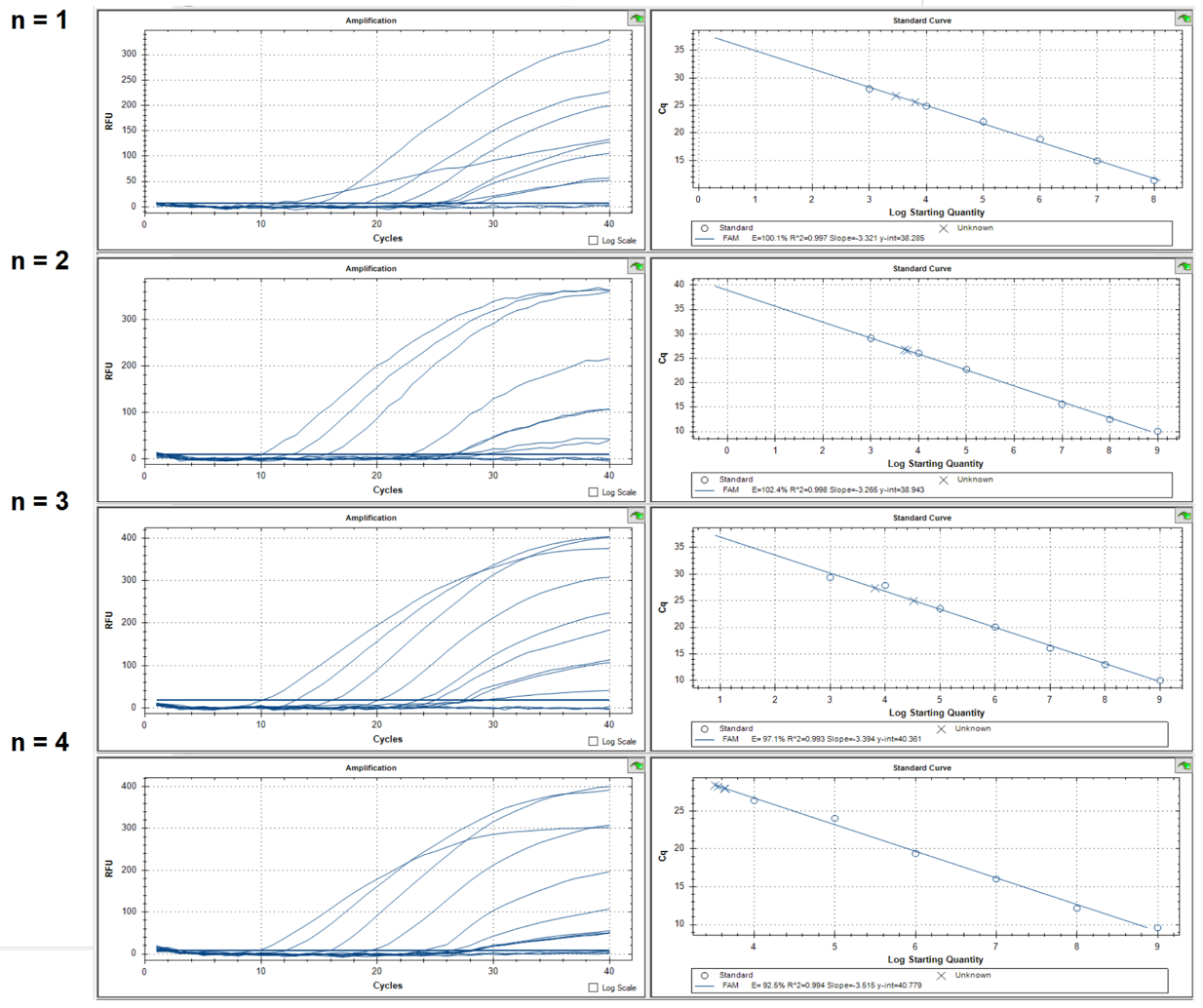

Supplement: S4 Fig — The standard curve was performed using 50 ng of gDNA, which was referred to as cell number 7575.5 cells, plus various amounts of amplicon in each copy number starting from 103 to 109 copies. By which the amount of amplicon in each copy number was calculated from the formula below. Numberofcopies=xng*6.0221*10^23molecules/moleN*660g/mole*10^9ng/g x: the amount of amplicon (ng) N: the length of the dsDNA amplicon Fifty ng of gDNA samples were used to amplify with RT-PCR. The starting quantity (SQ) from the amplification curve was used as a copy number of the sample to calculate the percentage of transduction efficiency (%TE), as shown in the formula below. %TE = copy number/cell number * 100 Primer forward sequence: CTGGCAGGAACATGTGGCGT Primer reverse sequence: CGTGGCTTGCCTCCCATCTC Probe: GCCGCTCCGCCGACGCACCA 50 ng of gDNA = 7575.5 cells Amplification curves and standard curves (n = 4). (TIF) [file pone.0284708.s005.tif]

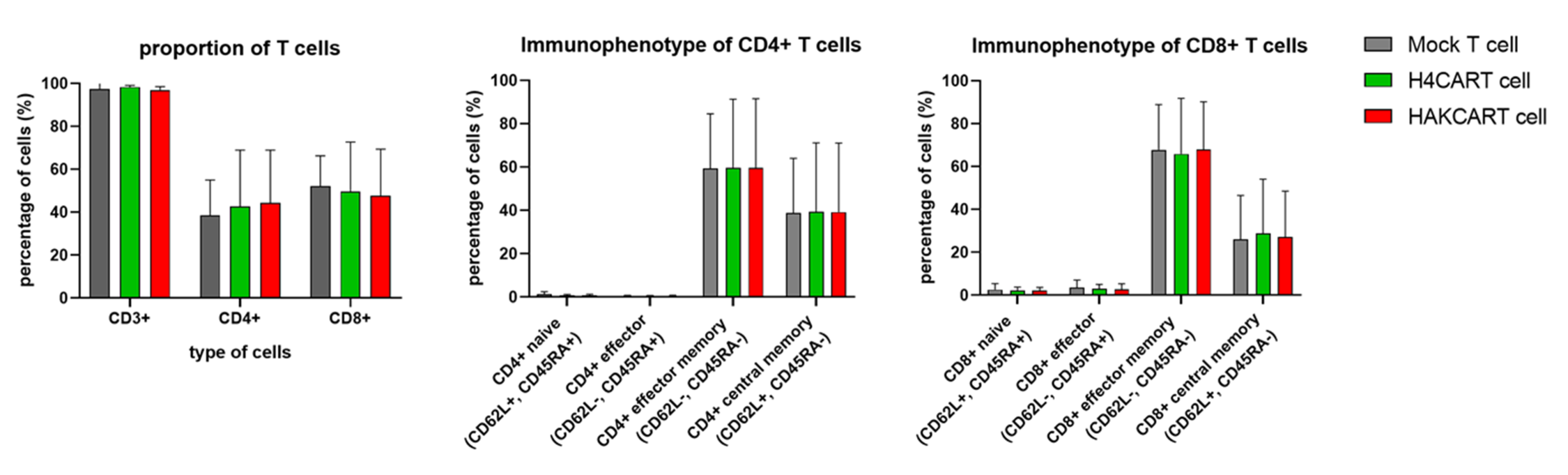

Supplement: S5 Fig — Each group of T cells consisted of almost 100% CD3+ T cells, divided into CD8+ T cells and CD4+ T cells. The proportion of CD8+ T cells was higher than that of CD4+ T cells. Each population was classified into four subpopulations: naïve T cells (CD62L+, CD45RA+), effector T cells (CD62L-, CD45RA+), effector memory T cells (CD62L-, CD45RA-), and central memory T cells (CD62L+, CD45RA-). Effector memory and central memory T cells in CD4+ and CD8+ T cells showed higher levels than naïve and effector T cells. (TIF) [file pone.0284708.s006.tif]

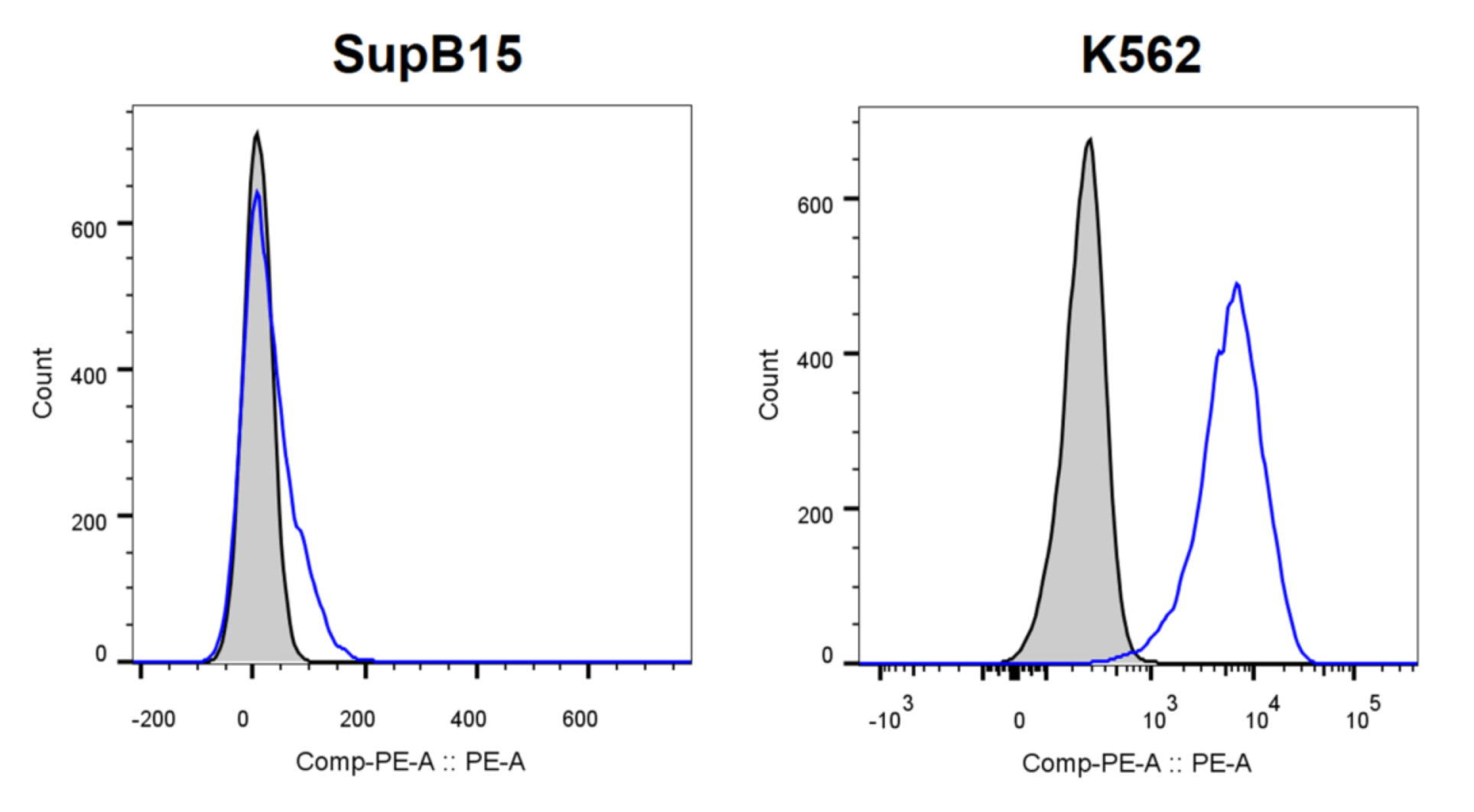

Supplement: S6 Fig — The CD30 antigen on the cell surface of healthy target cell lines (K562 and SupB15) was stained with PE anti-human CD30 antibody, and the expression was detected by flow cytometry. CD30 expresses at a consistently high level on the cell surface of K562 cells but not of SupB15 cells. The black-shaded histogram represented the unstain, and the blue showed CD30 staining. (TIF) [file pone.0284708.s007.tif]
